# Supplementary material for: Identification of Modifiable Risk Factors of Exacerbations Chronic Respiratory Diseases with Airways Obstruction, in Vietnam
Source: Int J Environ Res Public Health. 2022 Sep 4;19(17):11088. doi: 10.3390/ijerph191711088 (PMC9517880; doi:10.3390/ijerph191711088)

Supplementary Materials

PATIENT QUESTIONNAIRE.

I. Socio-economic status/demographics

1. Sex: 1. Male □ 2. Female □

2. Date of birth (dd/mm/yyyy): …………………………

3. Education: What is your highest degree?
3.1. Illiteracy □
3.2. Primary education (5-10 years old) □
3.3. Secondary education (11-18 years old) □
3.4. Post-secondary education □

4. Major/Occupation:
4.1. What is the main major/occupation in your life? ………………………
4.2. Please indicate the longest period you worked in this major/occupation (years):
4.3. Did you exposed to dust/fuels/chemicals when working on this major/occupation?
Yes □ No □

If you haven't any other major/occupation, please continue with question number 7.
If you have changed in other major/occupation, please continue with question number 6.

5. Current -major/occupation:
5.1. What is your current/occupation? ………………
5.2. Please indicate the longest period you worked in this /occupation (years):………
5.3. Did you exposed to dust/fumes/chemicals when working for this/occupation?
Yes □ No □

6. How much is your average income per month?
6.1. < 5 millions of VND
6.2. 5 – 10 millions of VND
6.3. > 10 millions of VND

7. Geographic origin: place of birth
A. Rural area □ B. City □
7.1. Please indicate the name of your place of birth: ……………… ………
7.2. Do you still live there? Yes □ No □
Yes, how long do you live there?......…………..years.
No, where do you live now? Rural area □ City □; from (year):………

8. General knowledge of chronic respiratory diseases (CRD):
8.1. Do you know the respiratory disease you have? Yes □ No □
8.2. In your opinion, can CRD cause death? Yes □ No □

8.3. In your opinion, CRD is as dangerous as cancer? Yes □ No □
8.4. In your opinion, CRD can improve with treatment? Yes □ No □

II. Smoking habits

1. Do you currently smoke?
   1. No, never □
   2. Yes, I do □
   3. No, I stopped smoking □ From (year):………..
   4. No, but I'm often exposed to second hand smoke □
2. What type of tobacco do/did you smoke?

		Number of unit/day	Number of year(s)
1. Cigarette	□	………………………..	……………………………
2. Cigar	□	………………………..	……………………………
3. Pipe	□	………………………..	……………………………

III. Indoor and outdoor pollution

1. Which of the following types of accommodation do you live in most of your life? (pictures in annexe)
   1. Non-tube house □ in ………year(s)
   2. Tube house □ in ………year(s)
   3. Rent house □ in ………year(s)
   4. Old appartment (built ≥ 20 years) □ in ………year(s)
   5. New appartment (built < 20 years) □ in ………year(s)
   6. Rural house □ in ………year(s)

2. A. How many people do you live with? ……. person(s)
B. What is your housing area? …….m^2
C. Does the house have windows that are open for ventilation? Yes □ No □

3. Indoor biological pollutants:
3.1. Mouldy ambiance Yes □ No □
3.2. Unpleasant smell Yes □ No □
3.3. Lots of dust Yes □ No □
3.4. Plants, flowers Yes □ No □
3.5. Pets Yes □ No □
Yes, please indicate the species: ……………………….
3.6. Birds Yes □ No □
3.7. Rats Yes □ No □
3.8. Cockroaches Yes □ No □

4. Indoor ventilation and chemical pollutants:
4.1. Use of air extractor Yes □ No □

4.2. Use of air conditionner Yes □ No □

4.3. Use of biomass stove Yes □ No □

Yes, for how many years: …………………….

4.4. Use of incense Yes □ No □

Yes, for how many years:.................................

4.5. Use of room spray products Yes □ No □

4.6. Use of volatile compounds (cleanse, wash) Yes □ No □

5. Distance from your accommodation to the public trafic:
   1. In front of the house (<10 meters) □
   2. Far from the house □ ……..meters

6. What floor do you live on?
   1. Basement □
   2. 0 - 2 □
   3. 3 – 6 □
   4. ≥ 7 □

7. Within the distance of <100 meters around where you live:
   1. Industrial park Yes □ No □
   2. Public entertainment center Yes □ No □
   3. Bus/coaches station Yes □ No □
   4. Landfill Yes □ No □

IV. Characterictics of chronic respiratory disease

1. Childhood and teenage period (0 - 17 years old) :

1.1. Gestational age at birth	□ Full-term	□ Premature	□ Unclear
1.2. Weight at birth	□ ≥ 2.5kg	□ <2.5kg	□ Unclear
1.3. Living environment	□ Unpolluted	□ Polluted	□ Unclear
1.4. Severe pulmonary infection	□ No	□ Yes	□ Unclear
1.5. Smoke exposure from parents	□ No	□ Yes	□ Unclear
1.6. Fumes exposure from fuel stove	□ No	□ Yes	□ Unclear
1.7. Diagnosis of asthma	□ No	□ Yes	□ Unclear

2. Have you had frequently the following symptoms within 3 last months?
   1. Dry cough Yes □ No □
   2. Cough with phlegm Yes □ No □
   3. Oppression Yes □ No □
   4. Wheezing Yes □ No □
   5. Shortness of breath Yes □ No □

2bis. If you frequently feel shortness of breath, please indicate the level of this symptom:

1. I only get breathless with heavy exercice □
2. I get shortness of breath when brisk walking on the flat road or walking up a slight hill □
3. I walk slower than people of same age on the level because of breathlessness or has to stop to catch breath when walking at their own pace on the level □
4. I have to stops for breath after walking about 100 meters or after few minutes on the level □
5. I'm too breathless to leave the house, or breathless when dressing or undressing □

3. Have you had any of the following situations within 12 last months?
1. Respiratory infections Yes □ ……time(s)/year; No □
2. Worsening of baseline symptoms Yes □ ……time(s)/year; No □

For each of above answers: if « Yes », continue question 4; if « No », continue question 5.

4. Which of the following treaments maked you feel better?
1. Bronchodilatators □
2. Antibiotics □
3. Corticosteroids □
4. Hospitalization/ICU □

5. When was the onset of your respiratory symptoms?
1. < age of 20 □
2. within 20 and 40 □
3. > age of 40 □

6. How were your respiratory symptoms?
1. Fluctuant □
2. Persistent □

7. Do you frequently have any of the following symptoms of allergies?
1. Runny nose Yes □ No □
2. Sinusitis Yes □ No □
3. Itchy red eyes Yes □ No □
4. Skin vesicules Yes □ No □
5. Rash Yes □ No □
6. Eyes/lip edema Yes □ No □

8. Do you frequently have any of the following symptoms of chronic rhinitis?
    1. Runny nose Yes □ No □
    2. Blocked nose Yes □ No □
    3. Itchy nose Yes □ No □
    4. Sneeze Yes □ No □

9. Does anyone in your family have the following problems?
    1. Asthma Yes □ No □
    2. Chronic obstructive pulmonary disease Yes □ No □

10. In your adulthood, have you ever been diagnosed with the following problems?
    1.Asthma Yes □ No □
    2.Chronic obstructive pulmonary disease Yes □ No □
    3.Chronis bronchitis Yes □ No □
    4.Pulmonary disease persist > 3 months Yes □ No □
    5.Emphysema Yes □ No □
    6.Pulmonary tuberculosis Yes □ No □

11. Do you have any of the following comorbidities?
    1.Hypertension Yes □ No □
    2.Diabetes Yes □ No □
    3.Cardiopathies Yes □ No □
    4. Gastro-intestinal diseases Yes □ No □
    5. Other(s) Yes □ No □
    Yes, please indicate the disease: ………………………….

Figure S1. Type of dwelling.

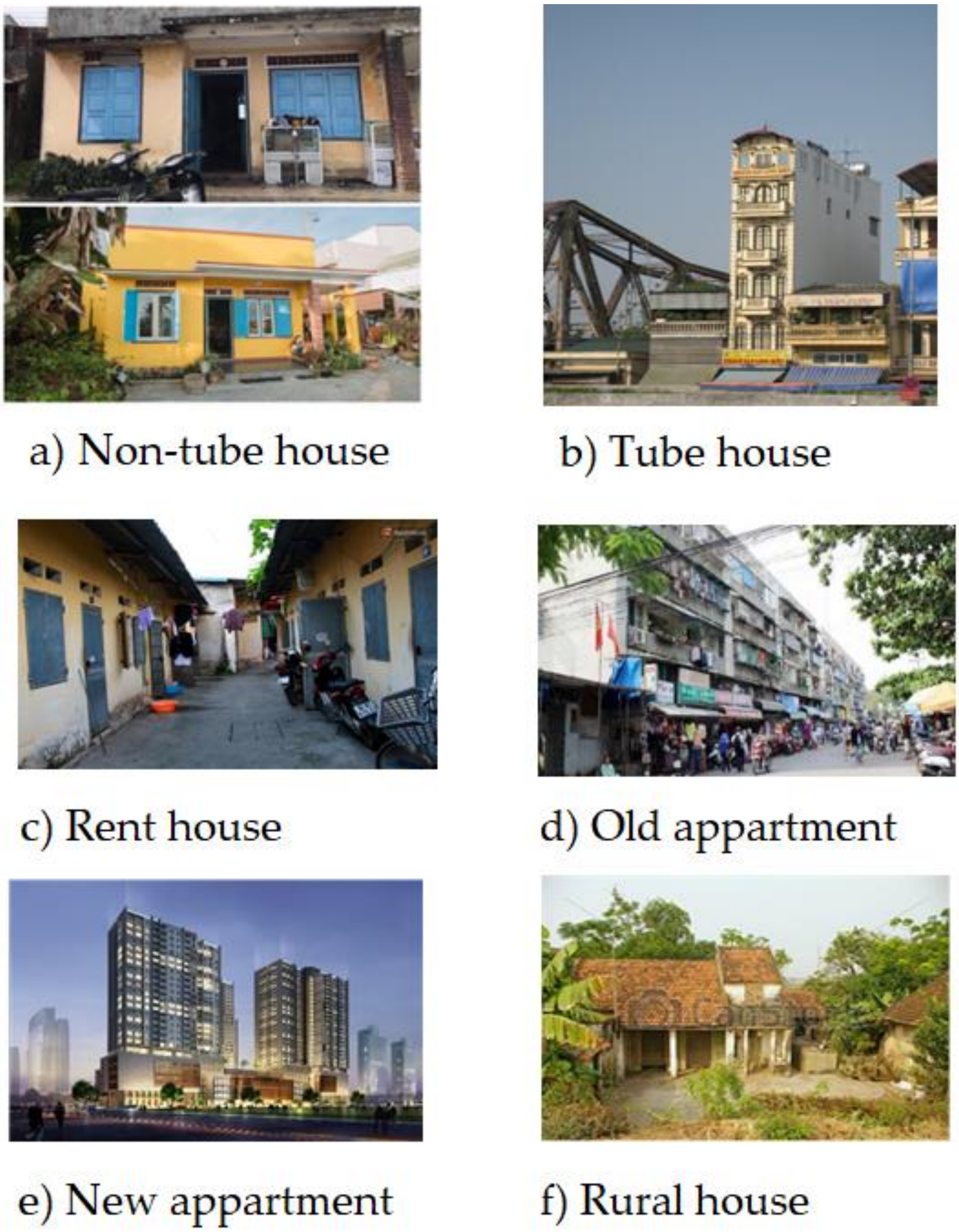

Supplement: Supplementary file 1 [file ijerph-19-11088-s001.zip › ijerph-1834390-supplementary.pdf]
